# Supplementary figures and images for: Structural prediction of chimeric immunogen candidates to elicit targeted antibodies against betacoronaviruses
Source: PLoS Comput Biol. 2025 Feb 5;21(2):e1012812. doi: 10.1371/journal.pcbi.1012812 (PMC11809852; doi:10.1371/journal.pcbi.1012812)

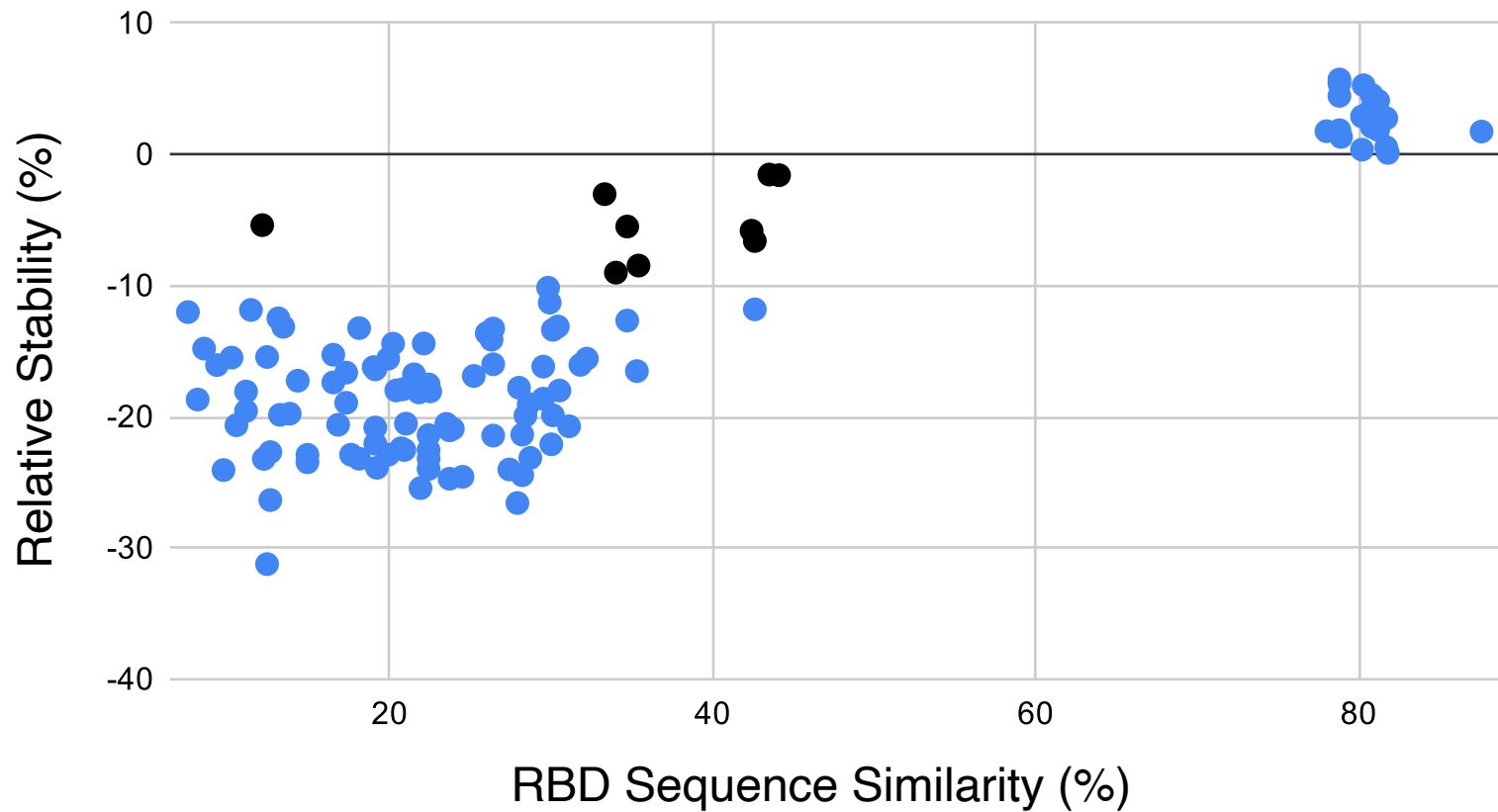

Supplement: S1 Fig — The relative stability score for each receptor-binding domain (RBD) chimera is plotted against the sequence similarity between the parental RBD sequence and SARS-CoV-2. There is less of a clear low-similarity, high-stability cluster than for the S1/S2 chimeras plotted in Fig 3. However, the 9 sequences with similarity <45% and relative stability>-10% were selected for further analysis by molecular dynamics simulation. (PDF) [file pcbi.1012812.s001.pdf]

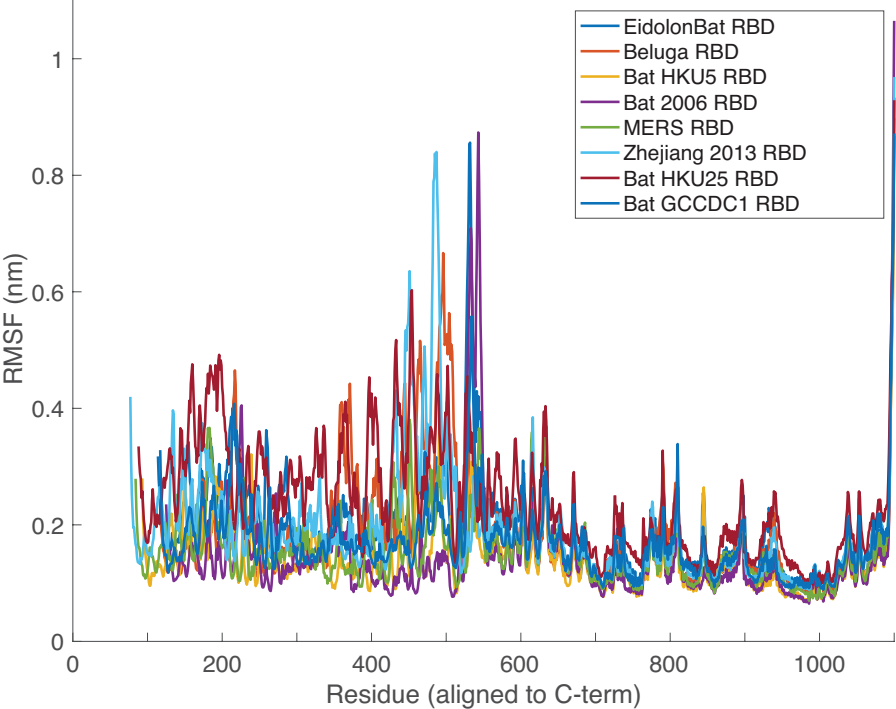

Supplement: S2 Fig — Root-mean-squared fluctuation (RMSF) values are plotted per residue for each of the simulated chimeras. Values were calculated on nanosecond intervals throughout the simulation trajectory. (PDF) [file pcbi.1012812.s002.pdf]

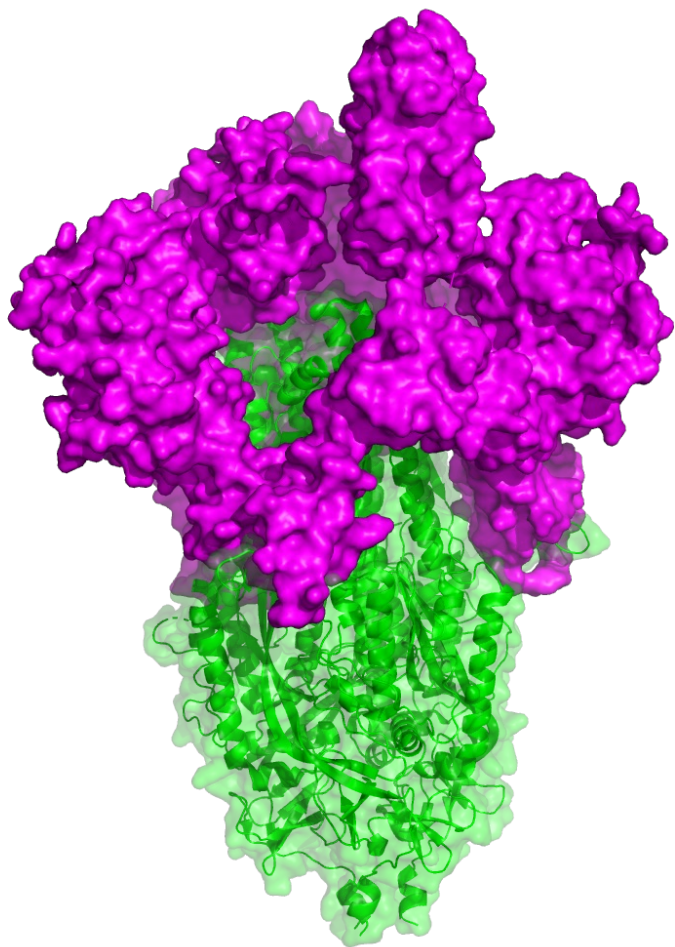

Supplement: S3 Fig — The model for SARS-CoV-2 Wuhan spike trimer from PDB 6VSB is rendered here with S1 in magenta and S2 in transparent green. Substantial interdigitation can be seen between the domains, with a large contact interface. The buried solvent-accessible surface area at the S1/S2 interface was calculated at 5154 Å2. This led us to the hypothesis that chimeras between low-similarity coronavirus spikes may frequently be unstable. (PDF) [file pcbi.1012812.s003.pdf]

a.

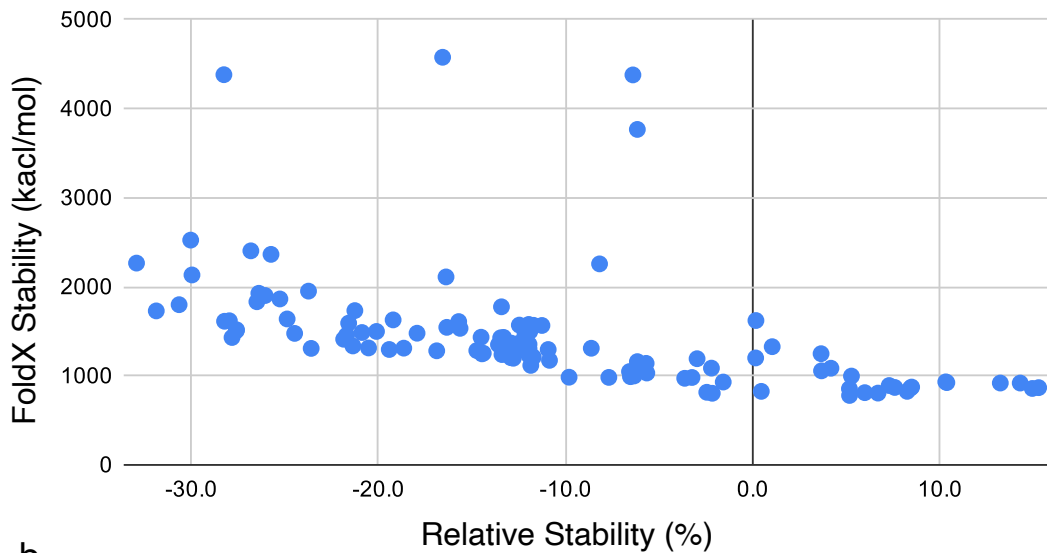

b.

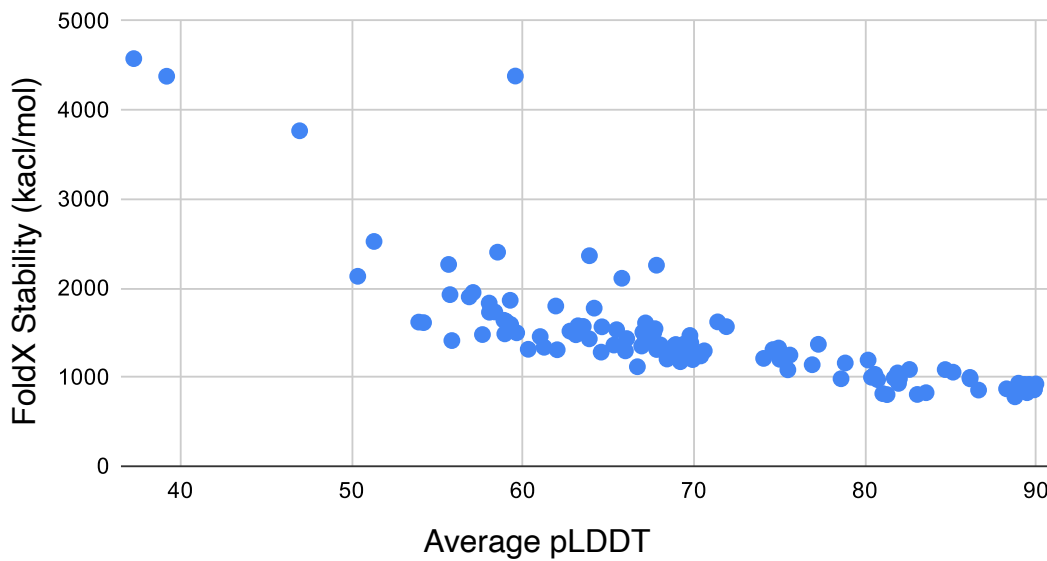

Supplement: S4 Fig — FoldX stability scores were calculated for each chimera and compared both to the relative stability metric (a) and the average pLDDT output from AlphaFold (b). Overall trends were similar, with a Spearman rank correlation of -0.89 between FoldX stability score and AlphaFold pLDDT and -0.78 between FoldX stability score and relative stability. Both plots show outliers with substantially higher FoldX stability. (PDF) [file pcbi.1012812.s004.pdf]

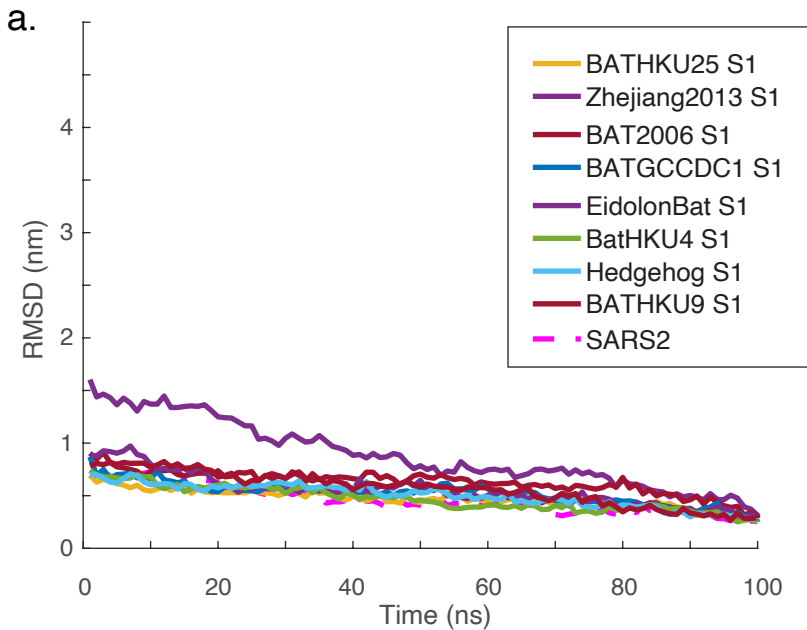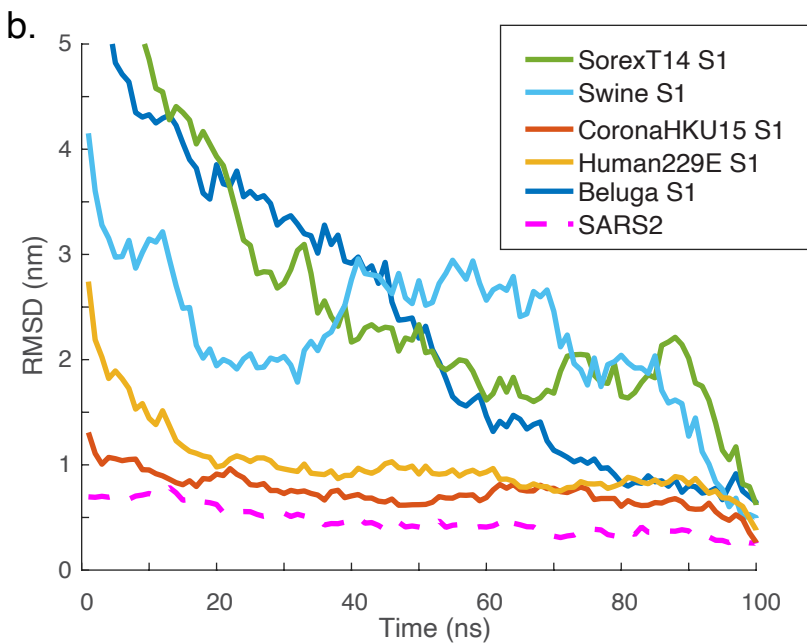

Supplement: S5 Fig — Panels (a-n) show the per-residue pLDDT from AlphaFold plotted against the RMSF from molecular dynamics simulations across the entire dataset of simulated S1 chimeras. Predicted high-stability chimeras are plotted in red, and predicted low-stability chimeras are plotted in magenta. The Spearman correlation coefficient between pLDDT and RMSF over the dataset is -0.69. (PDF) [file pcbi.1012812.s005.pdf]

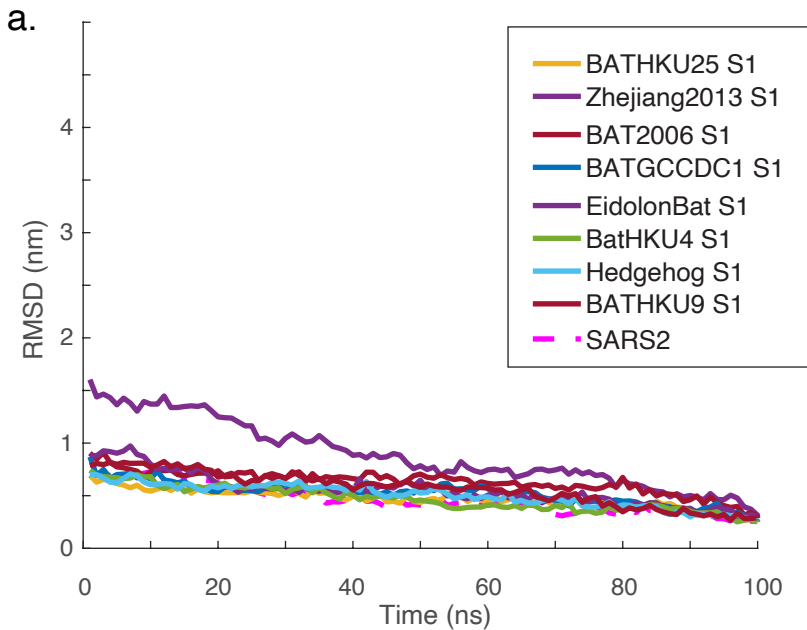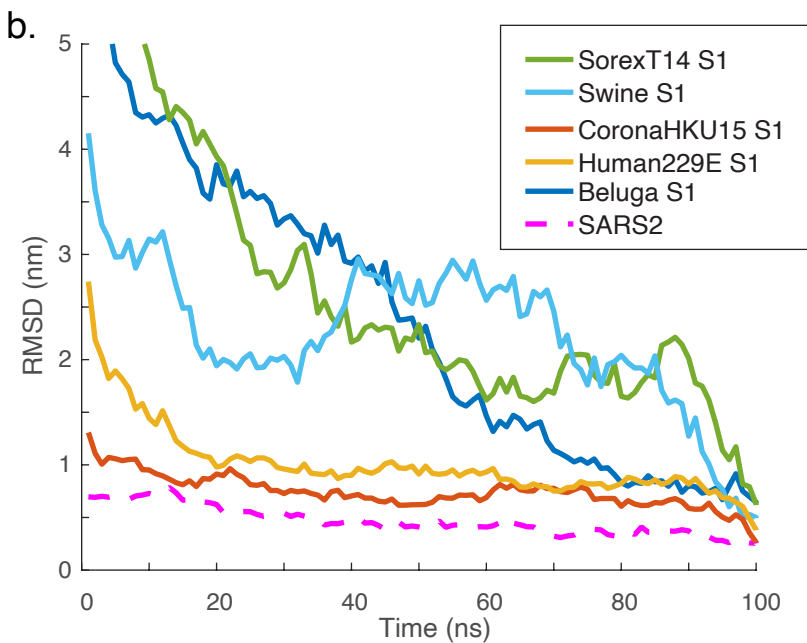

Supplement: S6 Fig — Root-mean-squared deviation values are plotted relative to simulated structures after 100 ns of simulation. Panel (a) shows the predicted-stable chimeras, and panel (b) shows the predicted-unstable chimeras. (PDF) [file pcbi.1012812.s006.pdf]
