# Supplementary material for: Structural prediction of chimeric immunogen candidates to elicit targeted antibodies against betacoronaviruses
Source: PLoS Comput Biol. 2025 Feb 5;21(2):e1012812. doi: 10.1371/journal.pcbi.1012812 (PMC11809852; doi:10.1371/journal.pcbi.1012812)
Supplement: S1 Table — (PDF) [file pcbi.1012812.s007.pdf]

Accession ID	Full Name	Short Name
QWN56273	Alphacoronavirus sp.	Alpha
YP_009199242	Alphacoronavirus 1	Alpha1
QDF43810	Coronavirus BtRs-AlphaCoV/YN2018	AlphaCorona
YP_009200735	BtRf-AlphaCoV/YN2012	AlphaCorona2012
AIA62242	BtMf-AlphaCoV/HuB2013-a	AlphaCorona2013
AIA62240	BtMf-AlphaCoV/GD2012-a	AlphaCoronaGD2012
QXT50810	Anser fabalis coronavirus NCN2	Anser
UBB42470	Jingmen Apodemus agrarius alphacoronavirus 1	ApodemusAlpha
UBB42425	Jingmen Apodemus agrarius betacoronavirus 1	ApodemusBeta
YP_009824998	Avian coronavirus	Avian
ADX59474	Rousettus bat coronavirus/Kenya/KY06/2006	BAT2006
ALJ94036	SARS-like coronavirus BatCoV/BB9904/BGR/2008	BAT2008
QHA24665	Rousettus aegyptiacus bat coronavirus 229E-related	BAT229E
ACU31051	Bat SARS Cov Rs806/2006	Bat806
ABG11963	Bat coronavirus A515/2005	BatA515
ABG11964	Bat coronavirus A527/2005	BatA527
ABG11965	Bat coronavirus A701/2005	BatA701
QLE11825	Bat alphacoronavirus	BatAlpha
YP_003858584	Bat coronavirus BM48-31/BGR/2008	BatBGR
QHA24724	Hipposideros pomona bat coronavirus CHB25	BATCHB25
YP_009273005	Rousettus bat coronavirus GCCDC1	BATGCCDC1
YP_008439202	Bat coronavirus CDPHE15	BatHE15
QHA24710	Hipposideros pomona bat coronavirus HKU10-related	BATHKU10rel
YP_006908642	Bat coronavirus HKU10	BatHKU10
ASL68941	Hypsugo bat coronavirus HKU25	BATHKU25
QCX35160	Tylonycteris bat coronavirus HKU33	BatHKU33
YP_001039953	Tylonycteris bat coronavirus HKU4	BatHKU4
YP_001039962	Pipistrellus bat coronavirus HKU5	BATHKU5
YP_001039971	Rousettus bat coronavirus HKU9	BATHKU9
ADX59495	Chaerephon bat coronavirus/Kenya/KY22/2006	BatKY22
ADX59458	Chaerephon bat coronavirus/Kenya/KY41/2006	BatKY41
ADX59451	Cardioderma bat coronavirus/Kenya/KY43/2006	BatKY43
YP_001876437	Beluga whale coronavirus SW1	Beluga
AYR18599	Betacoronavirus sp.	Beta
QWN56232	Betacoronavirus sp. RsYN03	Beta03
QWN56242	Betacoronavirus sp. RsYN04	Beta04
QWN56202	Betacoronavirus sp. RmYN05	Beta05
QWN56252	Betacoronavirus sp. RpYN06	Beta06
QWN56212	Betacoronavirus sp. RmYN07	Beta07
QWN56222	Betacoronavirus sp. RmYN08	Beta08

QWN56263	Betacoronavirus sp. RsYN09	Beta09
YP_009555241	Betacoronavirus 1	Beta1
AGC51116	Betacoronavirus BtCoV/KW2E-F93/Nyc_spec/GHA/2010	Beta2010
QJX58373	Coronavirus BtRt-BetaCoV/GX2018	BetaCorona
AIA62340	BtRf-BetaCoV/HuB2013	BetaCorona2013
QDF43815	Coronavirus BtRl-BetaCoV/SC2018	BetaCoronaSC2018
QRN68024	Betacoronavirus Erinaceus	BetaErin
YP_002308479	Bulbul coronavirus HKU11	Bulbul
ATI09449	Camel coronavirus HKU23	Camel
YP_009513021	Coronavirus HKU15	CoronaHKU15
YP_009380521	Coronavirus AcCoV-JC34	CoronaJC34
AWR88316	Deltacoronavirus sp.	Delta
QII89019	Bottlenose dolphin coronavirus	Dolphin
ADX59466	Eidolon bat coronavirus/Kenya/KY24/2006	EidolonBat
BBC54822	Falcon coronavirus UAE-HKU27	FalconHKU27
AYF53093	Feline alphacoronavirus 1	FelineAlpha
YP_009256197	Ferret coronavirus	Ferret
ABO88150	Bat coronavirus Fujian/773/2005	Fujian
YP_009755897	Canada goose coronavirus	Goose
YP_009513010	Hedgehog coronavirus 1	Hedgehog
QGA70692	Erinaceus hedgehog coronavirus HKU31	HedgehogHKU27
YP_005352863	Night heron coronavirus HKU19	HeronHKU19
BBC54832	Houbara coronavirus UAE-HKU28	HoubaraHKU28
YP_009194639	Human coronavirus 229E	Human229E
YP_173238	Human coronavirus HKU1	HumanHKU1
YP_003767	Human coronavirus NL63	HumanNL63
AZF86130	Alphacoronavirus Bat-CoV/P.kuhlii/Italy/206679-3/2010	Italy2010
AZF86118	Alphacoronavirus Bat-CoV/P.kuhlii/Italy/206645-41/2011	Italy2011
YP_009755890	Alphacoronavirus Bat-CoV/P.kuhlii/Italy/3398-19/2015	Italy2015
UBB42440	Jingmen Myotis chinensis alphacoronavirus 1	JingmenAlpha
QVN46559	Bat SARS-like coronavirus Khosta-1	Khosta1
QVN46569	Bat SARS-like coronavirus Khosta-2	Khosta2
UBB42462	Longquan Berylmys bowersi alphacoronavirus 1	LongquanAlpha
UBB42416	Longquan Niviventer niviventer betacoronavirus 1	LongquanBeta
QOE77336	Longquan Rl rat coronavirus	LongquanRat
YP_009336484	Lucheng Rn rat coronavirus	LuchengRat
YP_005352854	Magpie-robin coronavirus HKU18	Magpie
YP_009047204	Middle East respiratory syndrome-related coronavirus	MERS
QPP46979	Minacovirus mink/NLD/2020/NT_4	Minacovirus2020
YP_001718605	Miniopterus bat coronavirus 1	Minio
ADX59482	Miniopterus bat coronavirus/Kenya/KY27/2006	Minio2006
UBB42447	Jingmen Miniopterus schreibersii alphacoronavirus 1	MinioAlpha

YP_001718612	Miniopterus bat coronavirus HKU8	MinioHKU8
QHA24671	Miniopterus pusillus bat coronavirus HKU8-related	MinioHKU8rel
YP_009019182	Mink coronavirus 1	Mink
AVY53336	Alphacoronavirus Mink/China/1/2016	Mink2016
ADI80523	Mink coronavirus strain WD1133	MinkWD1133
AFD29244	Common moorhen coronavirus HKU21	MoorHKU21
YP_002308506	Munia coronavirus HKU13	Munia
YP_009824982	Murine coronavirus	Murine
YP_009199609	Myotis ricketti alphacoronavirus Sax-2011	Myotis
AHN92552	Mystacina coronavirus New Zealand/2013	Mystacina
YP_009201730	Nyctalus velutinus alphacoronavirus SC-2013	NyctalusAlpha
QLR06864	Pangolin coronavirus	Pangolin
QPN00063	Apodemus peninsulae coronavirus	Peninsuale
BBC54842	Pigeon coronavirus UAE-HKU29	PigeonHKU29
NP_598310	Porcine epidemic diarrhea virus	PorcineVirus
AXP20281	Quail deltacoronavirus	QuailDelta
BBC54852	Quail coronavirus UAE-HKU30	QuailHKU30
YP_005454245	Rabbit coronavirus HKU14	RabbitHKU14
YP_009113025	China Rattus coronavirus HKU24	RattusHKU24
YP_009199790	Rhinolophus ferrumequinum alphacoronavirus HuB-2013	RhinoAlpha
UBB42405	Jingmen Rhinolophus sinicus betacoronavirus 1	RhinoBeta
YP_001552236	Rhinolophus bat coronavirus HKU2	RhinoHKU2
QCX35167	Rhinolophus bat coronavirus HKU32	RhinoHKUU32
UAL80443	Swine acute diarrhea syndrome coronavirus	SADS
QZX47235	Sarbecovirus sp.	Sarbecovirus
QYC92806	Sarbecovirus RhGB01	SarbecovirusRhG
YP_001351684	Scotophilus bat coronavirus 512	Scotophilus512
ABO88151	Bat coronavirus Shandong/977/2006	Shandong
QPB10668	Shorebird deltacoronavirus	ShorebirdDelta
ATP66784	Sorex araneus coronavirus T14	SorexT14
AWV67107	Sparrow deltacoronavirus	SparrowDelta
YP_005352846	Sparrow coronavirus HKU17	SparrowHKU17
QEH62669	Swine enteric alphacoronavirus	Swine
QZQ78890	Tapir coronavirus 1044512-1	Tapir
YP_002308497	Thrush coronavirus HKU12-600	ThrushHKU12
QBG64657	Alphacoronavirus UKRn3	UKRn3
YP_009824974	Wencheng Sm shrew coronavirus	Wencheng
UBB42478	Wenzhou Suncus murinus alphacoronavirus 1	WenzhouAlpha
UBB42431	Wenzhou Pipistrellus abramus betacoronavirus 1	WenzhouBeta
YP_005352838	White-eye coronavirus HKU16	WhiteHKU16
YP_005352871	Wigeon coronavirus HKU20	WigeonHKU20
ALK02457	SARS-like coronavirus WIV16	WIV16

YP_009072440	Bat Hp-betacoronavirus Zhejiang2013	Zhejiang2013

Table S1. Sequences used in this study.
