# Supplementary material for: Structural prediction of chimeric immunogen candidates to elicit targeted antibodies against betacoronaviruses
Source: PLoS Comput Biol. 2025 Feb 5;21(2):e1012812. doi: 10.1371/journal.pcbi.1012812 (PMC11809852; doi:10.1371/journal.pcbi.1012812)
Supplement: S2 Table — (PDF) [file pcbi.1012812.s008.pdf]

Accession ID	Full Name	Short Name
AIA62240	BtMf-AlphaCoV/GD2012-a	AlphaCoronaGD2012
UBB42425	Jingmen Apodemus agrarius betacoronavirus 1	ApodemusBeta
ABG11963	Bat coronavirus A515/2005	BatA515
ABG11964	Bat coronavirus A527/2005	BatA527
ABG11965	Bat coronavirus A701/2005	BatA701
QCX35160	Tylonycteris bat coronavirus HKU33	BatHKU33
AGC51116	Betacoronavirus BtCoV/KW2E-F93/Nyc_spec/GHA/2010	Beta2010
QJX58373	Coronavirus BtRt-BetaCoV/GX2018	BetaCorona
AIA62340	BtRf-BetaCoV/HuB2013	BetaCorona2013
ATI09449	Camel coronavirus HKU23	Camel
UBB42478	Wenzhou Suncus murinus alphacoronavirus 1	WenzhouAlpha
UBB42431	Wenzhou Pipistrellus abramus betacoronavirus 1	WenzhouBeta

Table S2: Sequences Excluded due to AlphaFold Failures
