# Supplementary material for: Structural prediction of chimeric immunogen candidates to elicit targeted antibodies against betacoronaviruses
Source: PLoS Comput Biol. 2025 Feb 5;21(2):e1012812. doi: 10.1371/journal.pcbi.1012812 (PMC11809852; doi:10.1371/journal.pcbi.1012812)
Supplement: S3 Table — Names for chimeras selected for MD simulation are colored in red. (PDF) [file pcbi.1012812.s009.pdf]

Source for RBD (short name)	RBD Sequence Similarity (%)	Relative Stability (%)
Alpha	20.7	-22.4
Alpha1	12.6	-26.3
AlphaCorona	20.2	-14.4
AlphaCorona2012	30.5	-18.0
AlphaCorona2013	12.6	-22.7
Anser	26.3	-14.1
ApodemusAlpha	26	-13.6
Avian	20.4	-18.0
BAT2006	42.6	-6.6
BAT2008	80.2	0.4
BAT229E	22.4	-23.2
Bat806	81.2	4.1
BatAlpha	8.1	-18.7
BatBGR	80.2	2.9
BATCHB25	30.1	-19.9
BATGCCDC1	42.4	-5.8
BatHE15	17.6	-22.9
BatHKU10	19.1	-16.4
BATHKU10	14.3	-17.2
BATHKU25	33.3	-3.0
BatHKU33	19	-16.2
BatHKU4	34.7	-12.7
BATHKU5	34.7	-5.5
BATHKU9	42.6	-11.8
BatKY22	31.8	-16.0
BatKY41	22.4	-17.5
BatKY43	27.9	-26.6
Beluga	12.1	-5.4
Beta	23.5	-20.5
Beta03	80.8	4.5
Beta04	78.8	4.5
Beta05	78.8	5.4
Beta06	81.2	1.9
Beta07	80.8	3.5
Beta08	78.8	5.7
Beta09	78.8	1.8
Beta1	24.5	-24.6
BetaCoronaSC2018	81.7	0.5
BetaErin	29.8	-10.2

Bulbul	16.8	-20.6
CoronaHKU15	16.5	-15.3
CoronaJC34	26.4	-13.3
Delta	13.4	-13.1
Dolphin	22.5	-18.1
EidolonBat	44.1	-1.6
FalconHKU27	8.5	-14.8
FelineAlpha	14.9	-23.5
Ferret	19.1	-22.1
Fujian	21.9	-25.4
Goose	21.8	-18.1
Hedgehog	29.9	-11.3
HedgehogHKU27	34	-9.0
HeronHKU19	32.2	-15.6
HoubaraHKU28	22.4	-21.4
Human229E	29.5	-16.2
HumanHKU1	26.4	-16.0
HumanNL63	22.4	-22.5
Italy2010	16.5	-17.4
Italy2011	28.2	-21.4
Italy2015	13.8	-19.8
JingmenAlpha	17.3	-16.6
Khosta1	80.3	5.3
Khosta2	78	1.8
LongquanAlpha	31.1	-20.7
LongquanBeta	26.4	-21.4
LongquanRat	25.2	-16.9
LuchengRat	28.4	-19.9
Magpie	13.1	-12.5
MERS	35.4	-8.5
Minacovirus2020	11.1	-19.5
Minio	13.2	-19.8
Minio2006	14.9	-22.9
MinioAlpha	21	-20.5
MinioHKU8	10.2	-15.5
MinioHKU8rel	18.1	-23.2
Mink	11.1	-18.1
Mink2016	20.8	-17.9
MinkWD1133	12.2	-23.2
MoorHKU21	29.5	-18.6
Munia	21.5	-16.8
Murine	28.6	-19.0

Myotis	17.3	-18.9
Mystacina	9.7	-24.1
NyctalusAlpha	20.9	-22.5
Pangolin	87.6	1.7
Peninsuale	28	-17.8
PigeonHKU29	22.4	-24.0
PorcineVirus	12.4	-31.2
QuailDelta	12.4	-15.4
QuailHKU30	7.5	-12.0
RabbitHKU14	23.7	-24.7
RattusHKU24	28.7	-23.1
RhinoAlpha	19.9	-22.9
RhinoBeta	80.8	2.1
RhinoHKU2	30	-22.1
RhinoHKUU32	10.5	-20.6
SADS	28.2	-24.4
Sarbecovirus	81.7	2.8
SarbecovirusRhG	78.9	1.3
Scotophilus512	19.2	-23.9
Shandong	19.1	-20.8
ShorebirdDelta	9.3	-16.1
SorexT14	22.1	-14.4
SparrowDelta	18.1	-13.2
SparrowHKU17	19.9	-15.6
Swine	27.4	-24.0
Tapir	23.7	-21.0
ThrushHKU12	35.3	-16.5
UKRn3	30.1	-13.4
Wencheng	23.9	-20.9
WhiteHKU16	11.4	-11.9
WigeonHKU20	30.4	-13.1
WIV16	81.8	0.1
Zhejiang2013	43.5	-1.5

Table S3: Relative Stability Scores and Sequence Similarity for RBD Chimeras. Names for chimeras selected for MD simulation are colored in red.
