# Supplementary material for: Structural prediction of chimeric immunogen candidates to elicit targeted antibodies against betacoronaviruses
Source: PLoS Comput Biol. 2025 Feb 5;21(2):e1012812. doi: 10.1371/journal.pcbi.1012812 (PMC11809852; doi:10.1371/journal.pcbi.1012812)
Supplement: S4 Table — Names for predicted high-stability chimeras selected for MD simulation are colored in red, and names for predicted low-stability chimeras selected for MD simulation are colored in magenta. (PDF) [file pcbi.1012812.s010.pdf]

Source for S1 (short name)	S1 Sequence Similarity (%)	Relative Stability (%)
RhinoBeta	71.8	15.3
Beta03	72.2	14.9
Bat806	72.8	14.3
Beta07	71.8	13.2
Pangolin	78.6	10.4
Beta06	74.6	10.3
SarbecovirusRhG	68.5	8.5
BatBGR	69.1	8.4
Khosta2	70.6	8.2
BAT2008	67.8	7.6
Sarbecovirus	72.6	7.3
EidolonBat	35.9	6.7
Khosta1	69.8	6.0
Beta09	70.1	5.3
BetaCoronaSC2018	73.2	5.2
WIV16	72	5.2
Beta08	64.6	4.2
Beta05	64.6	3.7
BATGCCDC1	35.9	3.7
BAT2006	36.6	1.0
BATHKU25	32.1	0.5
Zhejiang2013	39.3	0.2
BATHKU9	38.1	0.2
BatHKU4	32.2	-1.6
Hedgehog	35.5	-2.2
Beta04	64.6	-2.2
BetaErin	35.1	-2.5
HumanHKU1	32.8	-3.0
MERS	32.4	-3.2
HedgehogHKU27	34.9	-3.6
Tapir	33.2	-5.7
Peninsuale	35.6	-5.7
Murine	35.4	-6.2
SorexT14	30.8	-6.2
LongquanBeta	33.4	-6.2
BATHKU5	33.2	-6.3
Dolphin	21.5	-6.4
LongquanRat	33.3	-6.5
RabbitHKU14	33.7	-6.6

Beta	35.3	-7.7
FelineAlpha	21	-8.2
Alpha1	24	-8.6
Beta1	32.2	-9.8
RhinoHKUU32	26.6	-10.9
Ferret	21.3	-10.9
BatAlpha	24.6	-11.3
PorcineVirus	24.4	-11.7
RattusHKU24	34.9	-11.7
MinkWD1133	24.6	-11.9
Italy2010	25.4	-11.9
RhinoAlpha	23.2	-12.0
MinioHKU8	25.8	-12.0
BatKY43	24.6	-12.0
Minacovirus2020	22.4	-12.0
Mink2016	22.7	-12.1
BATHKU10	25.2	-12.1
MinioAlpha	25.4	-12.2
HumanNL63	25.3	-12.3
Italy2011	23.8	-12.4
AlphaCorona2013	24.1	-12.5
Mink	22.9	-12.5
BatHKU33	25.2	-12.8
BATCHB25	25.4	-12.9
BatKY22	22.4	-12.9
BatHE15	25.6	-13.3
MinioHKU8rel	20.5	-13.3
Shandong	21.4	-13.4
Scotophilus512	25.6	-13.4
Fujian	24.2	-13.4
BatKY41	23.4	-13.5
Italy2015	25.2	-13.6
Minio	25.7	-14.4
NyctalusAlpha	23	-14.5
Alpha	25.5	-14.5
Myotis	26.7	-14.7
AlphaCorona	22.7	-15.6
JingmenAlpha	27.1	-15.7
Minio2006	19.8	-16.3
BatHKU10	26.8	-16.4
Beluga	23	-16.6
ShorebirdDelta	30.8	-16.9

Delta	32	-17.9
Mystacina	24.7	-18.6
ThrushHKU12	32.9	-19.2
BAT229E	21.7	-19.4
QuailDelta	30.9	-20.1
CoronaJC34	32.5	-20.5
LuchengRat	25.1	-20.9
UKRn3	30.7	-21.3
ApodemusAlpha	32.9	-21.4
WhiteHKU16	31.4	-21.6
Anser	29.6	-21.7
Wencheng	29	-21.9
Bulbul	27.6	-23.6
WigeonHKU20	29.9	-23.7
Munia	29.7	-24.5
Magpie	28	-24.9
PigeonHKU29	30.2	-25.3
Avian	27.5	-25.7
QuailHKU30	32	-26.1
SparrowHKU17	23.7	-26.4
HoubaraHKU28	29.9	-26.5
FalconHKU27	31.1	-26.8
SADS	30.3	-27.6
RhinoHKU2	33.7	-27.6
AlphaCorona2012	29.9	-27.8
Goose	29	-28.0
LongquanAlpha	26	-28.2
SparrowDelta	31.1	-28.3
HeronHKU19	28.3	-30.0
MoorHKU21	28.7	-30.0
Swine	33.7	-30.7
Human229E	31.7	-31.9
CoronaHKU15	31.7	-32.9

Table S4: Relative Stability Scores and Sequence Similarity for S1 Chimeras. Names for predicted high-stability chimeras selected for MD simulation are colored in red, and names for predicted low-stability chimeras selected for MD simulation are colored in magenta.
